# Artificial intelligence in echocardiography: detection, functional evaluation, and disease diagnosis

**DOI:** 10.1186/s12947-021-00261-2

**Published:** 2021-08-20

**Authors:** Jia Zhou, Meng Du, Shuai Chang, Zhiyi Chen

**Affiliations:** 1 The First Affiliated Hospital, Medical Imaging Centre, Hengyang Medical School, University of South China, 69 Chuanshan Road, Hengyang, 421001 China; 2 Institute of Medical Imaging, University of South China, Hengyang, China

**Keywords:** Artificial intelligence (AI), Echocardiography, Segmentation, Functional evaluation, Normalization

## Abstract

Ultrasound is one of the most important examinations for clinical diagnosis of cardiovascular diseases. The speed of image movements driven by the frequency of the beating heart is faster than that of other organs. This particularity of echocardiography poses a challenge for sonographers to diagnose accurately. However, artificial intelligence for detection, functional evaluation, and disease diagnosis has gradually become an alternative for accurate diagnosis and treatment using echocardiography. This work discusses the current application of artificial intelligence in echocardiography technology, its limitations, and future development directions.

## Highlights

1. Application of artificial intelligence (AI) in echocardiography is now widely studied, and AI technique has the potential to optimize the diagnostic potential of echocardiography.

2. Application of artificial intelligence in echocardiography is important in the following aspects: recognizing the standard section, cardiac cavity automatic segmentation, functional left ventricle assessment, and cardiac disease diagnosis.

3. Standardized data collection and image annotation are essential for artificial intelligence in echocardiography.

## Background

The application of artificial intelligence (AI) technology in cardiovascular imaging has become a research hotspot in recent years, as it may reduce treatment cost and help avoid unnecessary testing [1]. AI technology has been progressively applied for processing multiple modal images, such as auxiliary electrocardiograph diagnosis [2], cardiac computerized tomography (CT) detection [3], and radionuclide myocardial perfusion imaging [4]. In the diagnosis and treatment of heart diseases, AI techniques have been applied to electrocardiography, vectorcardiography, echocardiography, and electronic health records [5]. As a non-invasive imaging detection method for cardiac structure and functional evaluation, echocardiography technology has certain limitations. These include a long procedure time (more than 20 min, even if no abnormalities are detected), multiple measurement values that increase the duration complexity and user subjectivity, complex analyses during the evaluation, high standard of individualized assessments [6], high operator subjectivity, and wide observation ranges and distinctions among observers that persist even under standardized conditions. These limitations also lead to a high demand for medical specialist training in the field of echocardiography. In recent years, the application of AI for diagnosis and treatment using echocardiography has been shown to have the potential to solve these problems.

**Fig. 1:**
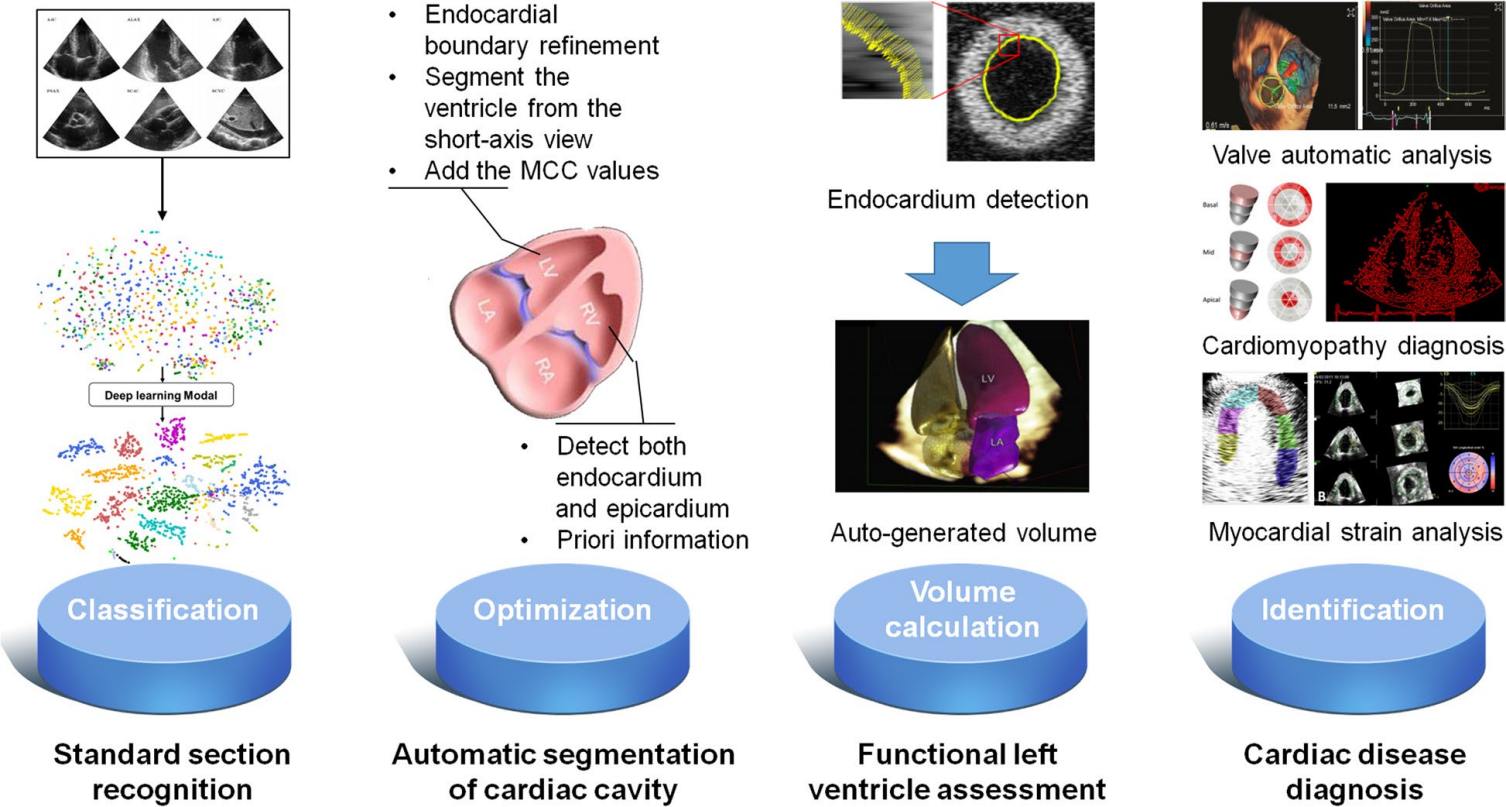
Application of artificial intelligence in echocardiography [12–15]

The integration of echocardiography and AI is not a brand new topic. Earlier cases of integrated application of echocardiography and machine learning can be traced back to 1978 when Fourier analysis was used to evaluate the waveform of anterior mitral leaflets via M-mode ultrasound. Studies have confirmed that this method had a remarkable impact on auxiliary diagnosis of mitral valve prolapse [7]. Machine learning is a significant AI method. Before deep learning was proposed in 2006, plenty of machine learning algorithms had been applied to echocardiographic evaluation of cardiac function, image optimization, and structural observation in the form of software or cutting edge technology, such as semi-automatic speckle tracking technology and the Simpson method. In early 2020, the Food and Drug Administration announced that it had authorized Caption Guidance software from Caption Health to be available for sale to collect data from echocardiographic images [8] (https://captionhealth.com/). The development of novel technologies, such as deep learning and neural networks, has effectively improved the efficacy of echocardiography [9], making standard section identification of cardiac anatomical structures, automatic recognition and segmentation of cardiac structures, cardiac functional evaluation, and auxiliary disease diagnosis faster and more accurate [10, 11] (Fig. 1). Although a series of articles published in high-level journals positively affirm the role of AI technology in diagnosis of echocardiography [16–18], there are remaining concerns, such as insufficient standardization of echocardiography, poor robustness, and insufficient generalization of the models in clinical applications. This review summarizes the application (Table 1) and advantages of echocardiography integrated with AI, analyzes the associated limitations, and systematically investigates the future trends of AI technology in echocardiography from the perspective of practical applications.

**Table 1:** Previous AI studies of echocardiography

	**Aim of study**	**AI algorithm or commercial technique**	**Detail method/software**	**Diagnostic Efficiency**^**a**^	**Reference**
**Section recognition/ image acquisition**	Automatic classification of cardiac views	AI algorithm	CNN	ACC = 98.3%	Ultrasound Med Biol, 2019 [19]
		AI algorithm	CNN	ACC = 97.8%	NPJ Digital Med, 2018 [12]
**Segmentation**	Left ventricular segmentation (provide accuracy improvement of the endocardial boundary recognition)	AI algorithm	Ant colony optimization	N/A	Biomed Mater Eng, 2014 [13]
		AI algorithm	Radial active contour method Snake	N/A	Comput Methods Programs Biomed, 2014 [20]
		AI algorithm	Iterative 3-D cross-correlation algorithm	N/A	Ultrasound Med Biol, 2014 [21]
		AI algorithm	Ant colony optimization	N/A	Biomed Mater Eng, 2014 [13]
	Right ventricular segmentation	AI algorithm	Sparse matrix transform/ wall thickness constraint feature	Dice = 90.8% (epicardial boundary), 87.3% (endocardial boundary)	Phys Med Biol, 2013 [22]
	Atrial and multi-chamber heart segmentation	AI algorithm	Active shape model/ fusion-imaging technology	Mean dice = 83.3% ~ 91.3% (LV)	Ultrasound Med Biol, 2015; IEEE Trans Ultrason Ferroelectr Freq Control, 2015 [23, 24]
**LV assessment**	Assessment of left heart function	Commercial technique	HeartModel (Philips)	*r* = 0.87 ~ 0.96/ *r* = 0.98	JACC: Cardiovascular Imaging, 2016; J Am Soc Echocardiogr, 2017 [25, 26]
		Commercial technique	AutoLV (TomTec Imaging system)	ICC = 1.0	J Am Coll Cardiol, 2015 [27]
		Commercial technique	AutoEF (BayLabs)	*r* = 0.95	Circ Cardiovasc Imaging, 2019 [28]
		AI algorithm	3D CNN	AUC = 0.92	J Am Soc Echocardiogr, 2020 [29]
		AI algorithm	PWC-Net	ACC = 97% ~ 98%	JACC Cardiovasc Imaging, 2021 [30]
		AI algorithm	Neural network	Intraclass correlation = 0.86 – 0.95 (AI and physician), 0.84 (novice using AI and physician)	Circ Cardiovasc Imaging, 2021 [31]
**Cardiac disease diagnosis**	Valvular heart disease	Commercial technique	Proximal isovelocity surface area (PISA)	Intraclass correlation coefficients = 0.96	Circ Cardiovasc Imaging, 2013; J Am Soc Echocardiogr, 2012 [32, 33]
		Commercial technique	Real-time 3D volume color-flow Doppler (RT-VCFD)	*r* = 0.93	Int J Cardiovasc Imaging, 2015 [34]
		Commercial technique	Mitral Valve Navigator (Philips)	N/A	Echocardiography, 2016 [35]
		Commercial technique	Anatomical Intelligence in ultrasound (AIUS)	ACC = 89%	J Am Soc Echocardiogr, 2016 [36]
		AI algorithm	Anatomical affine optical flow	Intra- and interobserver variability = 0.85 and 0.65	Int J Cardiovasc Imaging, 2019 [37]
		AI algorithm	2D/3D CNN	Sensitivity (Recall) = 67%, PPV = 74% ~ 77%	Nat Commun, 2021 [38]
	Cardiomyopathy	Commercial technique	Myocardial strain analysis	N/A	Circ J, 2019 [39]
		AI algorithm	Associative memory classifier	AUC = 89.2%	Circ Cardiovasc Imaging, 2016 [40]
		AI algorithm	Support Vector Machine	AUC = 77.8%	AMIA Annu Symp Proc, 2014 [41]
	Coronary atherosclerotic heart disease	AI algorithm	discrete wavelet transform and texture feature analysis	AUC = 90% ~ 99%	JACC Cardiovasc Imaging, 2019 [42]
		Commercial technique	EchoPAC PC (GE)	*r* = 0.67 ~ 0.99	Echocardiography, 2015 [43]
		AI algorithm	A specialised software for MCE quantification	Sensitivity = 77% Specificity = 94%	Ultrasound Med Biol, 2017 [15]
		AI algorithm	Texture analysis	Highest accuracy = 79%	IEEE, 2016 [44]
	Intracardiac masses	AI algorithm	Feed-forward neural network	Accuracy = 91%	Comput Med Imaging Graph, 2006 [45]
		AI algorithm	Gray level co-occurrence matrix-based features/ ANN (Classification)	Mean sensitivity = 0.955 Specificity = 0.970	J Ultrasound Med, 2014 [46]

*CNN* Convolutional neural network, *ACC* Accuracy, *AUC* Area under the curve, *ANN* Artificial Neural Network, *LV* Left Ventricular

^a^Diagnostic Efficiency: including recognition/diagnostic accuracies, correlation coefficient, PPV (positive predictive value), sensitivity (recall) and specificity, etc.

## Main text

### Standard section recognition with assistance of AI technology

The anatomical structure of the heart is complex, and sonogram genres are disparate. Therefore, it is particularly necessary to manage the recognition and functional evaluation of the cardiac structure in different models. However, there are numerous standardized sections of echocardiography. Patients without abnormalities have to undergo 10–20-section scans. Occasional slight angle differences among sections cause extreme difficulties in identification of various sections for physicians with insufficient experience and qualifications, resulting in failure to provide accurate and standardized analysis. It typically takes at least 1 to 2 years for a sonographer to grasp the basic echocardiography principles and methods of operation. In China, many grassroots regions cannot afford the labor cost of training echocardiography physicians. Therefore, two-dimensional (2D) ultrasound data sets from more than 500 patients and 7,000 videos were gathered from research performed in 2018. A classification model was then constructed using the method of convolutional neural networks by dividing them into seven groups of various cardiac views. The model’s classification accuracy reached 98% [19]. Likewise, researchers from the University of California, San Francisco used echocardiographic images from 267 patients to classify static and dynamic original images using deep learning approaches and to build an automatic section recognition model with classification criteria from 15 standard sections. The outcome showed that recognition accuracy achieved 97.8%, while physician recognition accuracy was approximately 70.2–83.5% [12]. Rapid standard section recognition with AI technology can somewhat shorten the evaluation time and enhance detection ability as well as novice accuracy. This technology has the potential to be applied in specialized non-echocardiography or emergency departments, which would advance operational efficiency. It can also be extended to the grassroots regions where echocardiography physician resources for standardized inspection and quality control are scarce.

### Functional left ventricle assessment with assistance from AI technology

Functional evaluation of the left ventricle is one of the most important and routine examination procedures in echocardiographic diagnosis. Functional evaluation indicators of the left ventricle systole include left ventricular ejection fraction (EF), left ventricular volume, left ventricular wall motion function, myocardial contractility and global longitudinal strain (GLS). EF is the most convenient and commonly used indicator for evaluating left ventricular systolic function. Since EF is a ratio when no segmental motion abnormality is present in the ventricular wall, EF can be determined using an M-mode chart by measuring the inside diameter ratio of the left ventricular end diastole to systole. A more precise approach for EF measurement is the biplane Simpson method, especially when segmental motion abnormalities such as myocardial infarction occur in the ventricular wall. The M-mode of certain left ventricular sections cannot represent the motion of the entire left ventricle. At this time, it is necessary to estimate the overall volume using the Simpson method. Regardless of the method employed, evaluations rely on visual observation and manual boundary tracing, while repeatability and accuracy depend on the physician’s experience [27]. The inter- and intra-observer variability can also be substandard when the functional left ventricle assessment is performed by a person.

Previous research on left ventricular function assessment with assistance of AI technology has been mainly based on automatic segmentation of the left ventricle and endocardial tracking technology [13]. At present, there are several commercial software packages that can achieve high-accuracy 2D and 3D echocardiography measurements, which further realize the automated assessment of the left heart function. One of the commonly used software is the Philips EPIQ series for transthoracic 3D echocardiography left ventricular cavity quantitative system HeartModel (Philips, Eindhoven, Netherlands), which utilizes an adaptive analysis algorithm [25, 26]. AutoLV (TomTec Imaging System, Germany) is an additional standard tracking system for left ventricular ejection fraction and longitudinal strain. Various clinical studies have shown that automatic software used to assess the ventricular volume and ejection fraction can provide accuracy that is similar to manual methods, which has a good correlation with cardiac MRI [27]. In spite of this, boundary identification is still prone to errors limiting accuracy. With the development of automated technique, some researchers have tried to reduce these errors by using evaluation methods without volume measurements. These algorithms mimicked what an experienced human eye and brain can do, instead of tracing the endocardial borders and calculating ventricular volumes [28, 29]. Since the analyses of GLS are time consuming and demand expertise, AI technique can help identify the standard apical views, perform timing of cardiac events, trace the myocardium, perform motion estimation, and measure GLS in less than 15 s. It was prove to have highly significant correlation with a conventional speckle-tracking application [30]. Furthermore, AI technology may help novices quickly acquire skills in quality diagnostic imaging to improve the inter- and intra-observer variability [31, 47]. For the prognosis, a novel multicenter research demonstrated that AI-based LV analyses were significant predictors of mortality, which is better than manual measurement. It could minimize variability of quantification of LVEF and LVLS [20]. AI can be a game-changer in this field, providing a reproducible LVEF evaluation that is independent of human observer.

### Automatic segmentation of cardiac cavity with assistance of AI technology

The shape and function of the four chambers of the heart (left/right ventricle, left/right atrium) are observed following the determination of the cardiogram section. If the heart morphology is affected by certain disease-related factors, the standard pressure and volume will change, resulting in cardiac chamber enlargement, compensatory wall thickening, and cardiac remodeling [21]. Therefore, it is important to obtain accurate segmentation of cardiac ultrasound images and understand the morphological changes for clinical diagnosis. Masses of echocardiography instruments are equipped with semi-automatic cardiac chamber segmentation software. Manual segmentation is tedious, time-consuming, and subjective. Hence, automatic and precise segmentation can decrease the occurrence of the above-mentioned problems and has favorable clinical values. At present, cardiac chambers are automatically segmented by recognizing the endocardial wall in 2D or three-dimensional (3D) images, which is common in segmentation of the left and right ventricles. During segmentation, the automatic evaluation and accurate measurement of parameters such as cardiac cavity size can also be accomplished.

#### Left ventricular segmentation

Left ventricular segmentation is popular in the automatic segmentation of the heart chamber. The accurate measurement of ejection fraction and evaluation of the movement in left ventricular myocardium can be achieved by segmenting the left ventricle. Two- and three-dimensional ultrasound methods are both widely used in the assessment of left ventricular segmentation. Compared to 2D ultrasound images, 3D-image resolution is lower. Manual processing and analysis are also extremely time-consuming. Fully automatic left ventricular segmentation based on ultrasound images remains a challenging task due to rapid and large-scale myocardial movements, respiratory interference, inconsistent motion between mitral valve opening and closing, in addition to inherent noise and artifacts in ultrasound imaging. Some methods have been proposed to address the above-mentioned issues. One solution evaluated the ventricular cavity function based on information such as variance to prioritize endocardial boundary refinement [13], followed by continuously tracking and measuring endocardial boundary deformation during the cardiac cycle. The other approach is to apply the radial active contour method Snake to segment the ventricle from the short-axis view instead of the long-axis view with a large deformation. This method is less influenced by noise and artifacts and has a more solid robustness [22]. In addition, for the automatic segmentation of the left ventricle in a 3D echocardiogram, segmentation performance can be effectively boosted by adding the maximum cross-correlation (MCC) values of the highest contrast between blood and heart tissues in the model. The MCC values help to obtain better recognition and segmentation effects when blood and myocardial-echo contrast is low [48]. A variety of methods used for accuracy improvement of the endocardial boundary recognition will prompt the precision of the automatic left ventricular segmentation.

#### Right ventricular segmentation

Compared to left ventricular segmentation, right ventricular segmentation presents a relatively difficult-to-resolve problem. These problems are related to the characteristics of the right ventricle and its wall, including complex crescent-shaped structure, presence of trabecular myocardium, thinner and weaker right ventricular walls, irregular endocardium shapes and edges on cardiogram images, and relatively poor image quality due to its behind-sternum, location leading to lung gas irruption and shadow of the sternum. These cause blurred ventricular wall echo and even disappearance of entire lateral walls in some images. However, accurate evaluation of right ventricular function is significant for functional analysis of the circulatory system, surgery selection of congenital heart disease (CHD), and prediction and evaluation of heart failure. The precise identification and segmentation of right ventricular ultrasound images can quickly and efficiently capture the diameter, area, and volume of the right ventricle, myocardial thickness, fractional area changes, as well as other indicators to provide more information for auxiliary clinical diagnostics. To overcome these issues, Qin et al. proposed an automatic segmentation framework based on the sparse matrix transform and introduced a wall thickness constraint feature. A positive segmentation result was acquired via detection of endocardium and epicardium at the same time when the lateral walls are blurred [23]. In addition, Bersvendsen et al. proposed and constructed a multi-chamber model based on how the chambers interact while performing the pumping function, which allows for coupled segmentation of endo- and epicardial borders of the left and right ventricle. The establishment of the multi-chamber model can allow for a complete clinical condition evaluation [24]. In general, when the image quality is poor, the precision of right ventricular segmentation is dependent on previously obtained information and correlation with other heart structures, such as myocardium, epicardium, and left ventricle.

#### Atrial and multi-chamber heart segmentation

Echocardiographic atrial segmentation has certain application values in minimally invasive interventional treatment of arrhythmia such as cardiac electrophysiology. For example, in radiofrequency ablation treatment of atrial fibrillation, 3D transesophageal echocardiography (3D-TEE) can be utilized to guide cardiac electrophysiological intervention (locating ablation points) in real time. However, there is a prerequisite that the changes in the atrial anatomical structure have to be real-time monitored for precise positioning [49]. Accordingly, Alexander et al. have applied an active shape model derived from computed tomography angiography (CTA) in a multi-chamber to segment the atrioventricle in a 3D-TEE image, improving segmentation accuracy in the left atrium, which previously had a poor segmentation performance. Furthermore, because 3D-TEE segmentation in the left atrium is vulnerable to scanning range restrictions, the team adopted fusion-imaging technology to merge the CTA and 3D-TEE images for construction of wide-view 3D-TEE images, which contributed to left atrial segmentation resolution and enhanced segmentation efficiency. This research has an important significance for cardiac surgery, where it can help to provide precise positioning for atrial fibrillation ablation [50, 51].

Although AI technology can effectively segment atrioventricular structures in echocardiography, recognition and segmentation precision needs to be advanced further due to interference of lung gas, ribs, and artifacts. Algorithm updates, such as multiple iterations, efficient search methods, particle filters, and online collaborative training approaches, combined with deep learning algorithms and multiple dynamic models [52] will further optimize echocardiographic segmentation performance.

### Cardiac disease diagnosis with assistance of AI technology

#### Valvular heart disease

Routine echocardiography can visually investigate cardiac valve shapes and activities. Its repeatability is not reliable due to subtle valve structure variations and wide heart motion ranges, as well as the intra- and inter-observer differences in recognizing valve stenosis, prolapse, calcification, and valve insufficiency. The mitral and aortic valves are the objectives of AI technology in cardiac valve evaluation, especially focusing on observation of valve morphology and regurgitation. Similarly, AI technique is helpful in the assessment of valvular heart disease. For example, automatic evaluation software for proximal isovelocity surface area (PISA) of mitral insufficiency can conduct an automatic measurement of mitral valve regurgitant orifice area and regurgitant volume to evaluate the severity of valve regurgitation. PISA for 3D echocardiography has a superior accuracy compared to 2D image analysis software, which has excellent consistency with transesophageal ultrasound and MRI measurements [32, 33]. Another study [34] used real-time 3D volume color-flow Doppler technique to quantify valve regurgitation volume, which also achieved high consistency in MRI measurements. In addition, valve morphology can be automatically analyzed by implementing automated measurements of the morphological mitral valve parameters, including 3D ring length and height, 2D area, commissural width, overlap width, 3D leaflet area, anterior and posterior leaflet angle, non-planar angle, prolapse, valve height, and volume. Previous research has confirmed that there is no significant difference between automatic and manual measurements [14]. This type of semi-automatic software has a favorable diagnostic value for valve morphology-related diseases such as mitral valve prolapses. It also enhances the non-experts’ accuracy in prolapse detection [35]. It can also be employed for intervalvular monitoring with advantages of consistency and repeatability [36].

Structural abnormalities in the congenital aortic valve, senile aortic valve calcification, and rheumatic fever are the common causes of aortic stenosis (AS). The method of aortic valve replacement is crucial for severe AS. Before valve replacement surgery, valve diameter measurement and areas in 2D echocardiography have been manually detected to evaluate the level of valve stenosis and provide a sufficient basis to determine the size of artificial valves. However, because of the dynamic changes in the position of aortic valves in vivo, 2D static image-based assessment is not only subjective but also only reveals the measurement results of one or two frames during the cardiac cycle. Some research studies [37] have adopted an automatic tracking algorithm based on anatomical affine optical flow for fast and automatic tracking of aortic valves and proximal end of the left ventricular outflow tract using 3D-TEE technology to optimize the measurement. It provides dynamic and accurate supporting information for preoperative planning of aortic valve replacement surgery, helping to improve the accuracy of valve evaluation and enhancing surgeon confidence.

#### Cardiomyopathy

Cardiomyopathy can be classified into two major categories of primary and secondary cardiomyopathy according to the cause. AI-assisted echocardiographic diagnosis of cardiomyopathy can be carried out via multiple imaging modes, such as 2D ultrasound, M-mode, and color Doppler. Based on accurate wall recognition and ventricular segmentation, automatic measurement of ventricular volume measurement, accurate assessment of cardiac function, and accurate visualization of myocardial movement through speckle tracking technology can be realized. The acquisition of these indicators helps to achieve rapid and precise detection of hypertrophic cardiomyopathy and cardiac amyloidosis. In the latest research, a human-interpretation-free machine learning pipeline based on the combination of ECG and echocardiography had been developed to detect cardiac amyloidosis. Multicenter study had confirmed that the artificial intelligence-enabled fully automated detection model outperformed interpretation by expert cardiologists in the diagnosis of cardiac amyloidosis [38]. On the other hand, speckle tracking imaging technology is widely used for the diagnosis of cardiomyopathy. Myocardial strain analysis is helpful for diagnosis of various types of cardiomyopathy [39]. Sengupta et al. created a machine learning algorithm based on speckle tracking technology images to distinguish between constrictive pericarditis and restrictive cardiomyopathy using an associative memory classifier (AMC). As an additional challenge, both of the diseases have similar ultrasonographic features, such as enlarged left and right atrium, relatively small ventricle, pericardial effusion, and widening vena cava. Clinical diagnosis emphasizes the changes in myocardial strain parameters. For example, strain values of left ventricular walls in constrictive pericarditis are significantly lower than those of ventricular septa, while restrictive cardiomyopathy does not have this feature. The study used AMC to demonstrate that analysis of the first 15 speckles tracking echocardiographic variables can classify diseases more accurately (area under the curve (AUC) of 89.2%). This method is better than the Doppler method alone (AUC of 82.1%) and the overall longitudinal strain (AUC of 63.7%), which obtained precision results. The study also used machine learning to automatically distinguish between male patients with hypertrophic cardiomyopathy and athlete cardiac physiological hypertrophy [40]. Due to the diversification of ventricular morphology in patients with dilated cardiomyopathy (DCM), segmentation of the left ventricular boundary (especially segmentation near the ventricle apex) is more complex than that of the normal left ventricle. To address the ventricular wall changes caused by DCM, Mahmood et al. [41] applied a support vector machine classifier to distinguish between normal and dilated left ventricles. Although the average classification accuracy of the study was only 77.8% (affected by cumulative errors), size evaluation accuracy of the left ventricle reached 87.2% and left ventricular boundary segmentation accuracy was 89.3%. The ROI region recognition accuracy was 92.5%, providing a research foundation for further development of reliable decision-making tools.

#### Coronary atherosclerotic heart disease

Coronary heart disease, also known as coronary atherosclerotic heart disease, is one of the most common coronary artery diseases, which is classified as a special type of cardiomyopathy. Echocardiography can assist in its diagnosis by visually observing myocardial movement and changes in cardiac morphology. AI technology can effectively improve subjectivity of conventional echocardiographic examination of coronary heart disease, increase detection systematicity, and help to better distinguish between normal and infarcted myocardial images [53]. For the ultrasound diagnosis of coronary heart disease combined with AI technology, experts have utilized the method of discrete wavelet transform and texture feature analysis [42], showing that the AI method using classifiers to automatically sort echocardiographic images can provide the characteristic parameters required for clinical diagnosis, as well as reduce the occurrence of complications. In terms of 3D ultrasound heart imaging, studies [43] have constructed myocardial infarction models in pigs and sheep and applied the EchoPAC PC (GE, Andover, MA, USA) program to analyze results. This method is a semi-automatic assessment of left ventricular quality and local strain values. In addition to traditional ultrasound examinations, myocardial perfusion can diagnose coronary artery disease by quantifying the infarct area via examination of myocardial contrast ultrasound. The AI method can also perform an automatic calculation of myocardial perfusion parameters and reduce human error [15]. Echocardiography can be integrated with machine learning to obtain a huge set of clinical data on echocardiographic changes. Machine learning can integrate these data into categories to assist physicians in accurately and rapidly diagnosing myocardial ischemia changes. In the prognosis of coronary heart disease, a previous research had develop a method based on texture parameters of native echocardiogram or contrast-enhanced acquisition to evaluate left ventricular function recovery 1 year after myocardial infarction. The highest rates of accurate prediction reached to 79% [44].

#### Diagnosis of intracardiac masses

In addition to the auxiliary diagnosis of the above-mentioned conventional diseases, AI technology can be applied to classify and recognize intracardiac masses (like left atrium/ear thrombosis, cardiac tumors and vegetation) [45]. For example, due to the complex and diverse anatomical structures of the left atrial appendage and the limited range of motion of the TEE probe, the resulting ultrasound artifacts may lead to misdiagnosis. Diagnosis through images of the left atrial (auricular) thrombosis depend on the patient’s anticoagulant drug plan. Sun et al. [46] performed transesophageal echocardiography on 130 patients with atrial fibrillation with image reconstruction. Subsequently, the study extracted the texture features of the gray-level co-occurrence image matrix and performed the classification using artificial neural networks. The model classification AUC was 0.932, which is much higher than sonographer's diagnosis, where AUC was 0.834. The study demonstrated that the Artificial Neural Network model can significantly improve TEE diagnosis of the left atrium (ear) thrombosis in patients with atrial fibrillation, which is conducive to early diagnosis and treatment [54].

### Clinical application limitations

In addition to black box effects, overfitting problems, ethical issues, and other common limitations associated with AI application in the medical field [55], AI echocardiography limitations also exist. First, echocardiography is not a bloodless mechanical operation. Communication between physicians and patient plays an indispensable role in disease diagnosis, while the current AI technology has not been able to perform human–computer interactions. In the future, there is a possibility that clinical work performed by a physician will be gradually substituted by AI, which will not in favor of further model optimization and sustainable development [56]. Based on our experiences, echocardiography and AI-integrated application, standardized data collection, and image annotation are essential. Due to its complexities and rapid heartbeat, the echocardiographic section standardization is more difficult compared to other organ section standardization, which may not conducive to the data collection in multi-center studies [57]. Furthermore, the difference of physiological and anatomical structures of the heart among races can not be ignore. It means that the model with a high accuracy on one specific dataset may not be feasible on another data set [58].Therefore, while accepting the assistance of this technology, sonographers should also consider how to utilize its best functions and determine what outcomes need to be adapted for patients.

### Conclusion and future outlook

Rich potential information contained in echocardiograms can be extracted and utilized by AI technology to boost the accuracy of diagnosis. At present, AI technology software is used to examine cardiac ultrasounds, such that the process no longer depends as much on the sonographer’s skills and experience, and with the added benefit of rapid disease diagnosis. The promising expansion of telemedicine with mobile and wireless technologies has produced unprecedented progress in the field of cardiovascular imaging [59]. With the implementation of remote echocardiography and application of robotic arms in 2014, as well as the realization of robot-assisted minimally invasive cardiac surgery, AI robotics technology also provides sufficient support to acquire, identify, and quantitatively analyze echocardiograms [60, 61]. With the continued development of AI technology, the future pattern of echocardiography will gradually transform to provide practical auxiliary assistance for patients.

## Data Availability

Data sharing is not applicable to this article as no datasets were generated or analysed during the current study.
